# The effect of relaxation techniques on edema, anxiety and depression in post-mastectomy lymphedema patients undergoing comprehensive decongestive therapy: A clinical trial

**DOI:** 10.1371/journal.pone.0190231

**Published:** 2018-01-05

**Authors:** Bahareh Abbasi, Navid Mirzakhany, Leila Angooti Oshnari, Ashkan Irani, Samaneh Hosseinzadeh, Seyed Mehdi Tabatabaei, Shahpar Haghighat

**Affiliations:** 1 School of Rehabilitation Sciences, Shahid Beheshti University of Medical Sciences, Tehran, Iran; 2 Shohada Hospital, Shahid Beheshti University of Medical Sciences, Tehran, Iran; 3 Biostatistics Department, University of Social Welfare and Rehabilitation Sciences, Tehran, Iran; 4 Occupational Therapy, School of Rehabilitation Sciences, Shahid Beheshti University of Medical Sciences, Tehran, Iran; 5 Quality of Life Department, Breast Cancer Research Center, Motamed Cancer Institute, ACECR, Tehran, Iran; Brown University, UNITED STATES

## Abstract

**Objectives:**

Lymphedema is sometimes accompanied by high degrees of anxiety and depression. This study aimed to assess the effects of relaxation techniques on the level of edema, anxiety and depression in women undergoing Comprehensive Decongestive Therapy (CDT).

**Design:**

This clinical trial compared two treatment methods in 31 women with post-mastectomy lymphedema, including 15 cases who received CDT and 16 who received RCDT (Relaxation plus CDT). The edema volume, anxiety and depression scores were compared at the first and last sessions of the first phase of the treatment and six weeks afterwards.

**Results:**

The edema, anxiety and depression scores were 63.6%, 54.1% and 65.5% in the RCDT group and 60.7%, 31.4% and 35.2% in the CDT group. There were significant differences between the two groups in terms of the reduction in depression (p = 0.024) and anxiety (p = 0.011) scores throughout the study. This significant relationship was due to the differences in the depression score in the 3^rd^ and 9^th^ weeks of the study between the two groups. Similarly, anxiety levels differed significantly between the two groups at the 9^th^ week of the study (P = 0.013).

**Conclusion:**

Relaxation techniques reduced the anxiety and depression scores and the volume of edema in the patients with lymphedema. The addition of this intervention to the therapeutic package for lymphedema patients requires further studies in terms of cost-effectiveness.

## Introduction

Breast cancer is the most common cancer in women throughout the world [1]. According to a report by the Iranian Cancer Registry, 7582 cases of breast cancer were identified in 2009 [2]. Iranian patients with breast cancer are five to ten years younger than the patients in other countries [1]. Breast cancer can cause fear, despair and mental trauma and challenge all the physical, psychological, social and spiritual aspects of the patient’s life from the moment of diagnosis [3]. Malamus (2010) showed substantially greater levels of anxiety and depression in women with breast cancer compared to healthy women [4].

Today, screening and early detection methods and advancements in therapeutic techniques have led to a significant development in the treatment of breast cancer [5]. The side-effects of breast cancer therapy, including lymphedema, increase in line with the higher survival of the patients [6]. Lymphedema consists of the accumulation of lymph in the interstitial spaces, mainly in the subcutaneous fatty tissues, caused by a defect in the lymphatic system. Lymphedema is a major complication of breast cancer and its treatment that can cause long-term physical and mental health consequences in patients [7]. Based on loco-regional and systemic therapies, highly-varying prevalence rates have been reported for breast cancer-related lymphedema, from 5% to 50%, with a rate of 10–30% in the first two years after surgery [8]. Lymphedema is a chronic medical condition that affects various functions, including daily activities, social and interpersonal relationships and occupational and domestic tasks, in 0.84–21.4% of breast cancer patients [9, 10]. The complication may also lead to a number of psychosocial issues, such as poor body image and reduced confidence in one’s body, physical inactivity, mental disturbances (e.g. anger, sadness and symptoms of depression), sexual problems, anxiety and social avoidance [11]. The psychological complications of lymphedema (e.g. depression and social isolation) decrease patients’ abilities and efficacy at work and home [12].

Comprehensive decongestion therapy (CDT) is an effective non-surgical technique for the treatment of lymphedema that has been recommended by the International Society of Lymphology [13]. This novel rehabilitation method involves manual lymphatic drainage (MLD), multi-layer compression bandaging, rehabilitation exercises and skin care [14]. Relaxation is a stress-reducing technique in which blood flow increases and the feeling of anxiety and worry diminish with the voluntary tensing and relaxing of different muscle groups within five to ten seconds [15]. Mac Clore used relaxation and exercise therapy in women with lymphedema and found that the patients’ arm flexibility, mood and quality of life improved in the follow-up three months later [16]. Laudan concluded that yoga can reduce edema and the associated symptoms of lymphedema in women with breast cancer [17]. Ghorbani reported yoga and Pilates to effectively improve the range of movement and reduce edema and pain in the upper limbs of women with breast cancer [18].

The mental problems associated with lymphedema are multifaceted and require intervention [9]. Edema volume reduction modalities alone cannot improve the poor psychological functioning of the patients efficiently [19]. Khodai et al. studied the use of non-pharmaceutical methods to reduce breast cancer complications. They proposed relaxation as an effective method for reducing cancer-induced psychological distress [20]. Given the high prevalence of breast cancer in Iran and the physical and mental consequences of post-mastectomy lymphedema, this study investigated the effects of relaxation techniques on the volume of lymphedema and levels of depression and anxiety in women with lymphedema undergoing CDT. The synergistic effects of relaxation and CDT are believed to help relieve the patients’ physical and psychological symptoms. These efforts can help develop practical multidimensional strategies for the more effective management of this complication.

## Materials and methods

This clinical trial was conducted on women with breast cancer-induced lymphedema attending Seyed Khandan Rehabilitation Clinic (Tehran, Iran) from January to May 2013. The participants were followed up until August 2013 and all of them had undergone partial or total mastectomy and completed the necessary adjunctive therapies (radiotherapy and chemotherapy) following a diagnosis of unilateral breast cancer. The inclusion criteria were unilateral lymphedema in the upper limbs (more than 200 cc of difference between the two sides), a post-surgery interval of at least one year, a minimum score of eight in each subscale of the Hospital Anxiety and Depression Scale (HADS), not having received treatment or relaxation techniques for lymphedema prior to the study and no history of severe psychological disorders requiring psychological pharmacotherapy. The patients meeting these criteria were invited to participate in the study. The research project was approved by the Ethics Committee of Shahid Beheshti University of Medical Sciences (Tehran, Iran) and registered at the Iranian Registry of Clinical Trials (IRCT) under the code IRCT2014111019891N1. Before beginning the study, the eligible women declared their willingness for participation by signing an informed written consent form.

Since contact and the exchange of information was likely to occur between the two groups during the first phase of the study (i.e. the first three weeks), randomizing the groups was not possible. Consequently, in order to increase the study rigor and reduce control contamination bias, all the patients underwent CDT over a six-month period. After recruiting the required sample and having the questionnaires filled out for them by a trained researcher, relaxation therapy was added to their CDT, and data collection for the RCDT (Relaxation plus CDT) group was achieved by a trained therapist. This strategy minimized the researcher’s interference in assigning subjects to the groups. Since the clinic maintained its routine care protocol throughout the entire study, randomization goals were largely satisfied and the patients were successfully allocated to the trial arms according to the discussed protocol.

**Fig 1:**
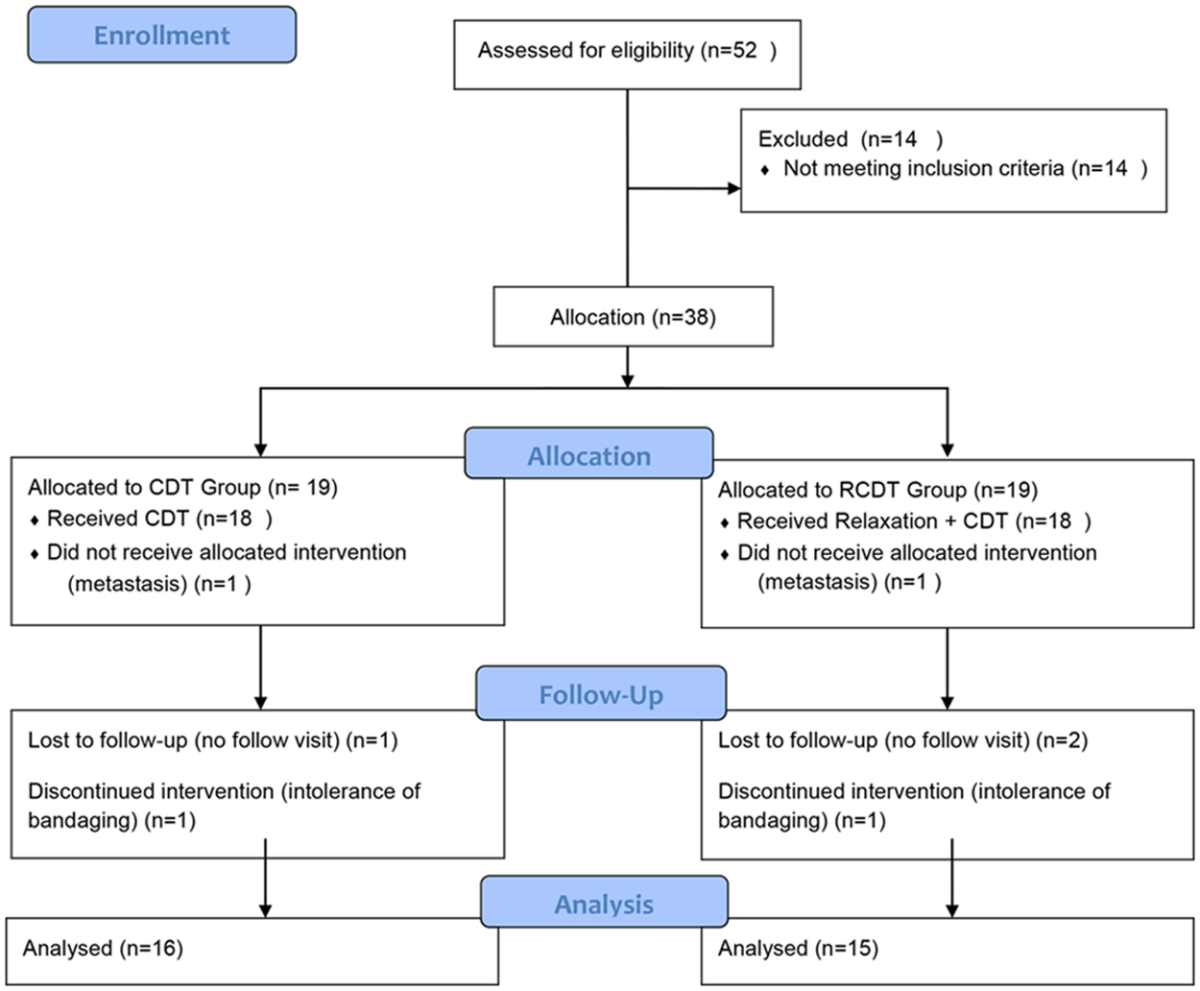
The flow diagram of the participants.

The mean difference in outcomes between the two groups was considered as the effect size. In accordance with previous studies [17] and considering a mean difference (standard deviation) of 0.17 (0.15) and α = 0.05 and β = 0.1, the sample size was determined as 16 in each group. To take account of potential sample loss, 38 patients entered the study. In the recruited sample, four patients in the CDT group and three in the RCDT group were excluded from the study because of incomplete follow-up or treatment and the need for other complementary breast cancer therapies. The remaining 31 women were studied in the RCDT (n = 15) and CDT (n = 16) groups. (Fig 1)

The demographic and clinical characteristics of the participants, including age, weight, height, marital status, education, type of breast surgery and number of excised and involved lymph nodes, were recorded in a checklist. A water displacement method (submerging the healthy limb and then the swollen limb in a water tank up to 2 cm below the armpit) [21] was performed to measure the edema. The Persian version of the HADS was also completed to assess anxiety and depression. The scale contained 14 items, including seven items on anxiety and seven on depression, and all the items were scored as 0–3. The validity and reliability of the scale had been previously confirmed by Montazeri et al. with a Cronbach's alpha of 0.78 for anxiety and 0.86 for depression [22].

The treatment was carried out in two phases. The first phase (acute phase) included 60-minute sessions held six days a week (excluding Fridays) for three consecutive weeks. In this phase, the CDT group was treated by MLD, multi-layer bandaging, rehabilitation exercises and skin and nail care training. The participants were trained to continue therapy during the second phase. In the second phase, therapy was performed at home using an educational brochure and CD for self-lymphatic drainage and exercises and by wearing an arm sleeve during the day, multi-layer bandaging at night, remedial exercises and continuing the skin and nail care.

In the RCDT group, the first phase involved 15 minutes of progressive muscle relaxation before each CDT session. During these sessions, the therapist instructed the patients on how to contract their different muscle groups for 5–7 seconds and then relax them for 10 seconds. This combined technique continued into the second phase of the treatment.

In both groups, edema, anxiety and depression were assessed at the beginning and the end of the first phase and in the second phase too (six weeks after the completion of the first phase) by a blinded person not involved in the treatment. The researcher made phone calls to the participants to follow up on their performance, monitor their treatment and answer any questions they might have once every two weeks.

The difference in outcomes between the first and third weeks of therapy compared to the initial value (volume at the beginning of the first week) was taken as the changes accomplished in the first phase. The patients were followed up between the 3^rd^ and 9^th^ weeks of the treatment. To define the changes in the follow-up phase, the difference in outcomes between the 3^rd^ and 9^th^ weeks of the treatment was divided by the 3^rd^–week’s end-values. The total changes in outcome was calculated based on the changes in outcome between the 1^st^ and 9^th^ weeks of the treatment.

The collected data were analyzed in IBM SPSS for Windows–version 19. The Chi-square test and Fisher’s Exact Test were used to compare the independent variables. The normality of the volume of edema and the level of anxiety and depression was examined using the Shapiro-Wilk test. None of these values had a normal distribution (P-value<0.05). The Mann Whitney U-test and the GEE (Generalized Estimating Equation) model and Bonferroni’s test for multiple comparisons were also used to assess the mean differences and changes in the outcome values over time (the level of statistical significance in Bonferroni’s test was 0.008). The level of significance in the two-tailed P-value was set as 0.05 for all the analyses.

## Results

Tables 1 and 2 present the demographic and clinical details of the RCDT and CDT groups. Participants’ age mean was 52.06 years in the CDT group and 55.4 years in the RCDT group. The mean body mass index (BMI) in the RCDT and CDT groups was 30.14 and 29.03 kg/m^2^, respectively. The interval between surgery and the initial assessment was 34.99 and 32.93 months in the two groups.

**Table 1:** A comparison of the quantitative (demographic and clinical) variables in the patients.

Variable	CDT Group (n = 16)		RCDT Group (n = 15)		P-Value
	Mean (±SD)	Range	Mean (±SD)	Range	
Age (year)	52.06 (±10.18)	37–75	55.47 (±11.51)	37–74	0.40
BMI (kg/m^2^)	29.03 (±4.31)	21.83–37.65	30.14 (±4.47)	24.46–39.14	0.48
Duration of Lymphedema	8.56 (±7.89)	1–29	10.76 (±14.08)	0.5–48	0.59
Time between Surgery and Onset of Lymphedema	32.93 (±25.63)	4–96	42.40 (±34.99)	8–95	0.39
Number of Excised Lymph Nodes	13.62 (±6.91)	1–25	12.13 (±4.51)	4–21	0.48
Number of Involved Lymph Nodes	5.18 (±5.16)	0–19	4.20 (±3.72)	0–12	0.54

**Table 2:** A comparison of the categorical (demographic and clinical) variables in the patients.

Variable	CDT Group (n = 16)	RCDT Group (n = 15)	P-Value
	No (%)	No (%)	
**Marital Status**			1*
Single	0 (0)	1 (6.7)	
Married	16 (100)	13 (86.6)	
Widowed/Divorced	0 (0)	1 (6.7)	
**Education**			0.95
Illiterate	1 (7.3)	1 (6.6)	
Primary School	4 (25)	6 (40)	
High School	9 (56.2)	4 (26.7)	
University	2 (12.5)	4 (26.7)	
**Dominant Limb**			0.08
Right	8 (50)	12 (80)	
Left	8 (50)	3 (20)	
**Involved Limb**			0.35
Right	8 (50)	5 (33.4)	
Left	8 (50)	10 (66.6)	
**Surgery Type**			0.68
MRM	13 (81.2)	13 (86.8)	
Breast Preservation	3 (18.8)	2 (13.2)	
**Chemotherapy**			0.33*
Yes	15 (92.7)	15 (100)	
No	1 (7.3)	0 (0)	
**Radiotherapy**			0.93
Yes	13 (81.2)	12 (80)	
No	3 (18.8)	3 (20)	

* Fisher’s Exact Test

The mean number of removed lymph nodes was 13 in the CDT group and 12 in the RCDT group. The mean number of involved lymph nodes was five and four in the two groups. According to Table 2, the majority of the patients in both groups were married. Moreover, 56.2% of the CDT and 40.0% of the RCDT group had a high school or lower level of education. Total mastectomy was the most common surgical procedure in both groups (81.2% and 86.6%). Radiation therapy and chemotherapy were conducted in 81.2% and 92.7% of the CDT and 80.0% and 100% of the RCDT group, respectively. The student’s t-test (Table 1) and the Chi-square test and Fisher’s exact test (Table 2) showed no significant differences in terms of the demographic and clinical details between the two groups.

According to Table 3, the Mann-Whitney test showed no significant differences between the two groups at the start of the treatment in terms of the volume of edema and the level of anxiety and depression. Anxiety differed significantly between the groups in the 9^th^ week (P-value = 0.041). Depression was significantly different between CDT and RCDT groups in the 3^rd^ and 9^th^ weeks (P-value<0.05).

**Table 3:** The median of the edema volume and anxiety and depression scores in the two groups during the study.

Variable	Time	CDT Group (n = 16)	RCDT Group (n = 15)	P-Value
		Median (IQR)	Median (IQR)	
**Edema Volume**	Start of Treatment	750 (904.2)	975 (562.5)	0.379
	3^rd^ week	375 (396.9)	471 (337.5)	0.495
	9^th^ week	262.5 (295.4)	324 (375.0)	0.470
**Anxiety Score**	Start of Treatment	11 (4)	11 (4.0)	0.861
	3^rd^ week	8.5 (4)	7 (5.0)	0.163
	9^th^ week	7 (4)	5 (3.0)	0.041
**Depression Score**	Start of Treatment	9.5 (4)	9 (2.0)	0.740
	3^rd^ week	8.5 (7)	4 (4.0)	0.021
	9^th^ week	6.5 (5)	3 (3.0)	0.003

The median reduction in the volume of edema in the first phase was 47.5% in the CDT group and 47.4% in RCDT group. This value was 25% and 31.2% in the follow-up period. As seen in Table 4, over the entire study period, the percentage of reduction in the volume of edema was slightly greater in the RCDT group than in the CDT group (64.3% vs. 62.4%). The Mann-Whitney test, however, did not suggest a significant difference between the two groups at any stage.

**Table 4:** The percentage of reduction in edema volume and anxiety and depression level in the first phase (1^st^ to 3^rd^ week), follow-up phase (3^th^ to 9^th^ week) and entire study period (1^st^ to 9^th^ week).

Variable	Time	CDT Group (N = 16)		RCDT Group (N = 15)		P-Value
		Median	IQR	Median	IQR	
**Edema Volume**	**First Phase**	47.5	20.8	47.4	25.6	0.830
	**Follow-Up Phase**	25.0	22.3	31.2	16.9	0.384
	**Entire Study**	62.4	12.8	64.3	17.1	0.800
**Anxiety**	**First Phase**	33.3	56.6	41.7	39.4	0.151
	**Follow-Up Phase**	10.0	25.0	20.0	30.3	0.345
	**Entire Study**	27.3	41.4	60.0	30.3	0.008
**Depression**	**First Phase**	11.1	61.1	59.6	43.2	0.041
	**Follow-Up Phase**	11.1	30.0	35.4	50.5	0.046
	**Entire Study**	33.3	61.5	71.0	23.0	0.007

In the control group, the median percentage of reduction in the anxiety scores was 33.3%, 10% and 27.3% in the first phase, the follow-up phase and the total study period. In the RCDT group, these rates were respectively 41.7%, 20% and 60%. The difference between the two groups was significant in the entire study period (P = 0.008). In addition, the two groups showed significant differences in the percentage of reduction in the depression scores in the first phase (P = 0.041), follow-up phase (P = 0.046) and the entire study period (P = 0.007); (Table 4).

The GEE was fitted for assessing the trend of changes in the edema, anxiety and depression scores in the two groups. Table 5 presents the results of the model.

**Table 5:** The results of the GEE model and Bonferroni’s test for multiple comparisons as a comparison of the changes in the edema volume and anxiety and depression level in the two groups.

Variable	Source	P-Value	Repeated Comparisons (P-Value)	Repeat* Group Comparisons (P-Value)	
				CDT Group	RCDT Group
**Edema Volume**	Repeat	<0.001			
	Group	0.726			
	Repeat * Group	0.684			
**Anxiety**	Repeat	<0.001			
	Group	0.154			
	Repeat * Group	0.011	Start (1.000)	Start - 3^rd^ week (0.015)	Start - 3^rd^ week (<0.001)
			3^rd^ week (1.000)	3^rd^ - 9^th^ week (1.000)	3^rd^ - 9^th^ week (0.003)
			9^th^ week (0.192)	Start - 9^th^ week (<0.001)	Start - 9^th^ week (<0.001)
**Depression**	Repeat	<0.001			
	Group	0.002			
	Repeat * Group	0.024	Start (1.000)	Start - 3^rd^ week (0.239)	Start - 3^rd^ week (<0.001)
			3^rd^ week (0.128)	3^rd^ - 9^th^ week (0.138)	3^rd^ - 9^th^ week (0.045)
			9^th^ week (0.005)	Start - 9^th^ week (0.002)	Start - 9^th^ week (<0.001)

Repeat * Group: Interaction of repeat and group variables

In the assessment of the changes in the edema volume throughout the study, only the difference of repeat variable was significant between the two groups. Bonferroni’s test for multiple comparisons showed that the mean edema volume differed significantly at baseline and in the 3^rd^ and 9^th^ weeks (P <0.008).

For the anxiety outcome, the group variable was not significant but the repeat variable was significant. Bonferroni’s test showed that the mean anxiety level differed significantly at baseline and in the 3^rd^ and 9^th^ weeks. Furthermore, the interaction of repeat and group was significant (P-value = 0.011). The multiple comparisons test showed that the mean anxiety score did not differ significantly between the two groups in any of the three repeats. In all the groups, however, the mean anxiety level differed significantly at baseline and in the 3^rd^ and 9^th^ weeks.

The changes in the depression score were significant in the repeat, group, interaction repeat and group variables. The multiple comparisons showed significant changes in the depression score in the two groups in the 9^th^ week (P = 0.005) of the study. Also, in the CDT group, the depression score differed significantly at baseline and in the 9^th^ week (P = 0.002). In the RCDT group, the depression score differed significantly at baseline and in the 3^rd^ week (P<0.001) and at baseline and in the 9^th^ week (P<0.001).

**Fig 2:**
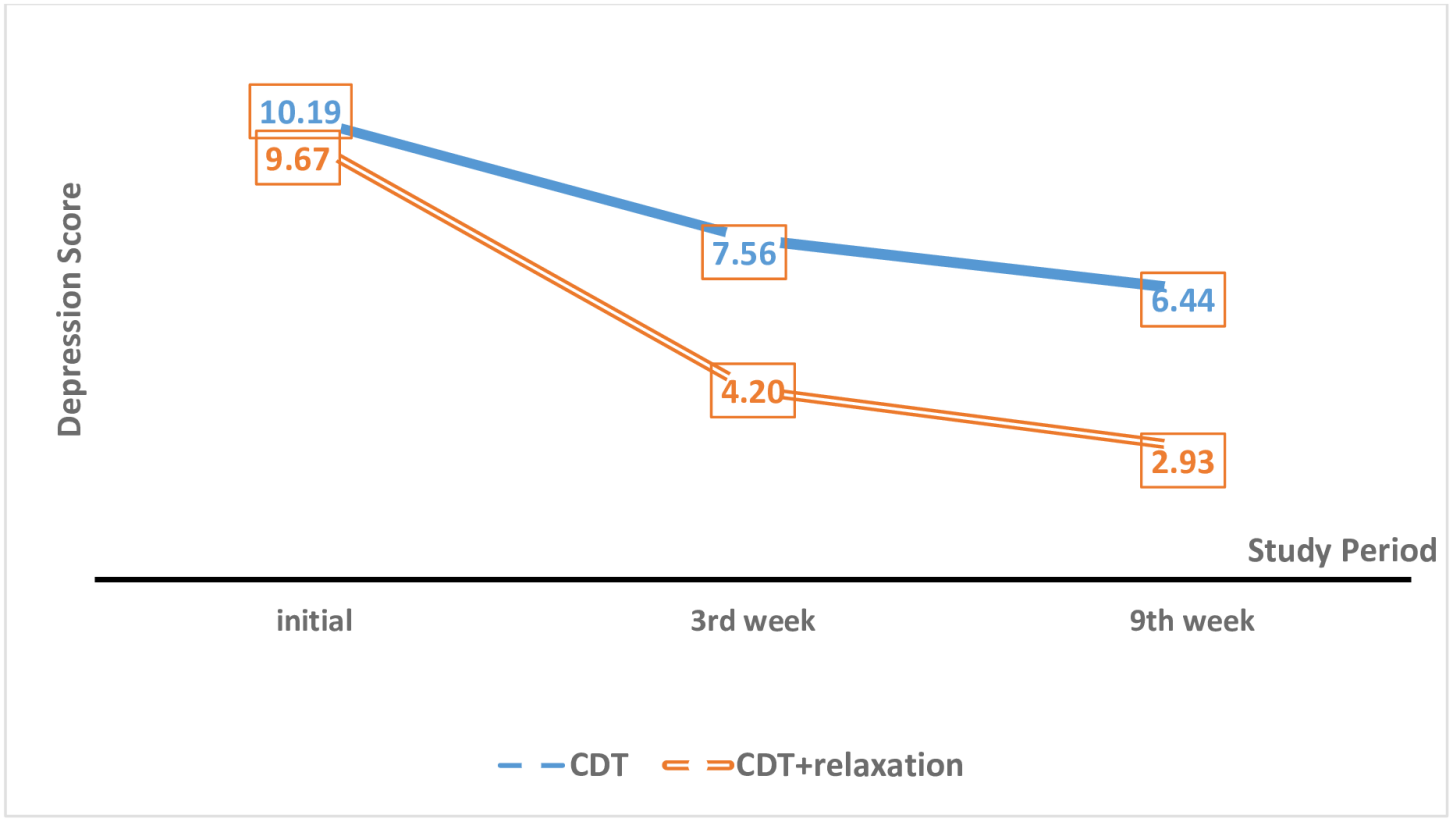
Differences in the depression score between the CDT and RCDT groups at baseline and in the 3^rd^ week and 9^th^ weeks.

The mean volume of edema and anxiety and depression scores showed a downward trend in both groups over time (Fig 2). According to the GEE repeated-measures model, there was a significant difference in the mean changes in anxiety (P = 0.011) and depression (P = 0.024) scores between the two groups.

## Discussion

In a lymphedema clinic, mental problems happen to be a multifaceted issue that requires interventions. Based on the present findings, compared to CDT alone, the simultaneous application of daily CDT and progressive muscle relaxation over nine weeks leads to a greater percentage of reduction in depression, anxiety and edema volume in women with lymphedema. The significant overall trend of changes in anxiety and depression during the study showed the effectiveness of relaxation techniques on these variables in the RCDT group.

The present study was conducted on 31 women with breast cancer-induced lymphedema. These participants were similar to the participants of other studies in terms of their clinical and demographic characteristics. The mean age of the CDT and RCDT groups was 52.0 ± 10.8 and 55.0 ± 11.5 years in the present, which is similar to the study by Khoshnazar et al. (53.0 ± 9.5 years) [1]. The mean BMI was 29.0 ± 4.3 kg/m^2^ in the CDT group and 30.0 ± 4.4 kg/m^2^ in the RCDT group. According to Muchin, 40% of individuals with lymphedema have a BMI between 25 and 30 kg/m^2^ [23]. Moreover, the majority of the present participants were married in both groups, which was also the case in the other studies [24, 25]. The present article has been published in Persian but with a different analysis method [26] and learning about the epidemiological characteristics of these patients can be helpful for Iranian therapists.

In this study, the percentage of reduction in the depression score was significantly higher in the RCDT group (69.5%) than in the CDT group (35.1%). In other words, adding progressive muscle relaxation to CDT caused a greater reduction in depression compared to CDT alone. Muscle relaxation is known to exert positive effects on patients’ psychological problems. In a study by Michael Anthony et al., ten weeks of cognitive-behavioral stress management intervention reduced depression (moderate type) and improved optimism according to the three-month follow-up [27]. Similarly, a study by Sadjad-Khodaee et al. in Iran showed a cognitive-behavioral intervention to reduce depression [20]. In these studies, relaxation was a major part of treatment. Considering the relatively high prevalence of depression and anxiety in breast cancer patients and the importance of controlling such complications along with the continued treatment of lymphedema, further extensive studies are warranted to determine the long-term efficacy of relaxation techniques in these patients.

In the present study, a greater volume of reduction in the edema volume was achieved through RCDT compared to CDT alone, but the difference was not significant; however, the reduction achieved in depression and anxiety differed significantly between the two groups. This finding confirms the hypothesis that the improvement in the volume of edema might be related to more important factors than anxiety and depression severity, such as type of lymphedema and the treatment method. A larger sample size and a consideration of potentially contributing factors (such as different levels of anxiety, depression or edema) may help gather a more accurate estimation of the combined effect of these outcomes in future studies.

The present findings showed a reduction in anxiety scores in both groups (54.1% in the RCDT group and 31.4% in the CDT group). This difference was observed in all the three stages of the study, but was only significant in the overall study period. In a study by Mohammad Pedram et al., cognitive-behavioral group therapy using relaxation and visualization reduced depression and anxiety in women with breast cancer [28]. According to studies by Tatro [29] and Bridge [19], relaxation reduces psychological distress and improves the mood in breast cancer patients. In 2008, Strauss et al. showed that a rehabilitation program involving MLD, exercise therapy, relaxation training and massage can improve the HADS scores in patients with breast cancer after a six-month follow-up [30]. Relaxation reduces stress, anxiety and depression in women with breast cancer, and relaxation exercises (progressive muscle relaxation) comprise a substantial part of cognitive-behavioral stress management programs [31]. The addition of this intervention to the treatment protocols used for highly-stressed patients with lymphedema is therefore recommended.

Although the two groups were not significantly different in terms of the reduction in the volume of edema, a higher reduction was observed in the RCDT group. Laudan [17] and Ghorbani [18] showed that yoga can decrease the volume of edema and increase the range of movement in patients with lymphedema. McClure et al. concluded that three months of relaxation and exercise therapy improve the quality of life and mood in women with lymphedema [16]. Nevertheless, since these studies did not use CDT in the treatment of lymphedema and did not measure edema using the water displacement method, a precise and firm conclusion cannot be drawn. Besides, the different effects of other relaxation techniques, such as yoga or other exercises, should also be taken into consideration.

A major advantage of this study was recruiting the participants on two different occasions. Despite the absence of a randomization process, this design minimized the chance of contact and the exchange of information between the two groups and in turn reduced the effect of the potential confounding factors and eliminated ethical concerns about not providing the control group with relaxation training. Since no significant differences were detected in the frequency distribution of the demographic and clinical characteristics between the two groups, a successful random allocation is assumed. Furthermore, the proper and effective application of the relaxation techniques besides the increasing participants' adherence to intervention were ensured through the researcher’s constant supervision. The costs of adding relaxation to CDT and the need for training personnel should be considered in future cost-effectiveness studies. The measurement of outcomes by an independent person with no involvement in the treatments given to the groups minimized the chance of observer bias. The results therefore seem to be generalizable to lymphedema clinics that provide such standards of treatment. The allocation of time for the completion of the questionnaires, the increased treatment period due to relaxation and the incomplete treatment in some patients were among the limitations of this study, which might have affected the patients' treatment tolerance. The researchers tried to control these limitations by assisting the patients and replacing the withdrawn subjects. The integration of relaxation techniques into the treatment of lymphedema patients appears to be beneficial, and including such techniques in the educational courses offered to students and therapists working in health centers may help improve the patients’ quality of life.

## Conclusion

The results confirm the effectiveness of progressive muscle relaxation in reducing anxiety and depression and its mild effects on the edema volume in patients with lymphedema undergoing CDT. Examining the efficacy and cost-effectiveness of different combinations of psychological and lymphedema treatments, especially in people with psychological disorders, requires more extensive studies.

## Supporting information

S1 Appendix The trial protocol in Persian. (PDF)

S2 Appendix The trial protocol in English. (PDF)

S1 Text Dual publication. (PDF)

S2 Text CONSORT checklist. (PDF)
