# Supplementary material for: The effect of relaxation techniques on edema, anxiety and depression in post-mastectomy lymphedema patients undergoing comprehensive decongestive therapy: A clinical trial
Source: PLoS One. 2018 Jan 5;13(1):e0190231. doi: 10.1371/journal.pone.0190231 (PMC5755759; doi:10.1371/journal.pone.0190231)
Supplement: S1 Appendix — (PDF) [file pone.0190231.s001.pdf]

پروتکل مطالعه

**عنوان طرح**	بررسی تأثیر تکنیک های تن آرامی بر میزان حجم ادم لنفاوی، اضطراب و افسردگی درزنان دارای ادم لنفاوی تحت درمان با احتقان زدایی ترکیبی
**واژگان کلیدی**	تکنیک های تن آرامی،احتقان زدایی ترکیبی، ادم لنفاوی، اضطراب، افسردگی
**ارائه دهنده**	دانشکده توانبخشی دانشگاه علوم پزشکی شهید بهشتی
**نوع مطالعه**	کارآزمایی بالینی
**هدف مطالعه**	تعیین تاثیر تکنیکهای تن آرامی بر میزان حجم ادم ادم، اضطراب و افسردگی در زنان دارای ادم لنفاوی ناشی از سرطان پستان تحت درمان با احتقان زدایی ترکیبی در ابتدا، 3 و 9 ماه بعد از درمان
**جامعه مورد مطالعه**	در این تحقیق، جامعه پژوهش در ، کلیه زنان دارای ادم لنفاوی اندام فوقانی (غیر از نوع خفیف) ناشی از درمان های سرطان پستان که دارای نمره 8 و بالاتر در تست HADS هستند.<br>جامعه هدف: کلیه زنان مبتلا به سرطان پستان یک طرفه که منجر به برداشت پستان (ماستکتومی) شده و تحت درمان های اولیه قرار گرفته باشند. جامعه تحت مطالعه: بیماران مبتلا به ادم لنفاوی ناشی از درمان های سرطان پستان، مراجعه کننده به کلینیک لنف ادم مرکز فیزیوتراپی سیدخندان می باشند.
**روش نمونه گیری و محاسبه اندازه نمونه**	در این پژوهش روش نمونه گیری غیر احتمالی در دسترس خواهد بود ،به نحوی که محقق در مرکز حضور خواهد داشت و کلیه بیماران مبتلا به ادم لنفاوی (با نظر پزشک) و واجد شرایط معیار های ورود ، که در زمان تحقیق به این مرکز مراجعه می کنند به عنوان نمونه انتخاب خواهند شد.<br>نمونه در این تحقیق با استفاده از تحقیق انت لودان که در این زمینه انجام شده و استفاده از تفاوت میانگین(0/17) و انحراف معیار(0/15 میانگین) با احتساب(α=0/05) و(β=0/1) به دست آمد. که تعداد نمو نه محاسبه شده 32 نفر است .16 نفر در گروه کنترل و 16 نفر در گروه درمان تن آرامی قرار میگیرند.
**روش بررسی**	

بعد از ارائه معرفی نامه رسمی به مسئولین محیط انجام مطالعه و جلب موافقت آن ها ،بیماران مبتلا به ادم لنفاوی یک طرفه ناشی از درمان سرطان پستان که معیار ورود را کسب کرده اند، وارد مطالعه می شوندو پرسشنامه مربوط اطلاعات دموگرافیک را تکمیل می نمایند .<br>قطعا در این طرح تصادفی کردن دو گروه امکان پذیر نیست، زیرا در طی 3-2هفته درمان دو گروه قطعا با هم تماس پیدا می کنند و احتمال انجام تن آرامی و تاثیر آن در گروه شاهد هم وجود دارد.<br>لذا ابتدا در یک مقطع زمانی بیماران تحت CDTتنها قرار می گیرند بعد از تکمیل حجم نمونه گروه اول و تکمیل پرسشنامه های لازم، درمان تن آرامی به CDT اضافه می شود.<br>جهت تعدیل اثر متغیر زمان و نوع درمان بر پیامدهای مورد مطالعه، سعی می شود نمونه ها در شرایط زمانی و درمانی مشابه انتخاب شوند. در صورت لزوم گروه دوم از نظر متغیرهای احتمالی موثر بر پیامد مانند حجم ادم و سن با گروه شاهد نسبتا مشابه انتخاب شوند.<br>با توجه به اینکه مطالعه کارآزمایی بالینی تصادفی شده انجام نمی شود ولی با Match کردن خصوصیات دو گروه سعی می شود شرایط دو گروه را خیلی به هم نزدیک و قابل مقایسه نماییم.<br>علت ارائه درمان ترکیبی پس از درمان CDT تنها این است که این احتمال وجود دارد که تراپیست با آشنا شدن با تکنیک تن آرامی و احیانا مشاهده نتایج مثبتی از آن در بیمارانی که قرار است تحت CDT تنها قرار گیرند، ناخودآگاه بخشی از این تکنیک را آموزش دهد یا اجرا نماید.<br>ارزیابی های صورت گرفته قبل از شروع مداخله شامل؛اندازه گیری میزان حجم ادم لنفاوی با استفاده از تانک حجم سنج و اضطراب و افسردگی با تکمیل پرسشنامه اضطراب و افسردگی بیمارستانی(HADS) است،که این اندازه گیری توسط شخص دیگری غیر از محقق انجام می شود.<br>این پژوهش توسط 2 نفر که اشنایی کامل به درمان های مورد نظر دارند انجام می شود.به منظور عدم سوگیری در نتایج به دست امده ؛در این تحقیق ارزیابی ها توسط فرد دیگری غیر از پژوهشگر انجام می شود.	
اجرای تحقیق شامل دو فاز درمانی است:بعد از ارزیابی های صورت گرفته بیماران وارد فاز اول درمان می شوند.	**نحوه اجرای تحقیق**

بیماران در هر دو گروه در فاز اول برنامه CDT که شامل چهارجزء(ماساژ،بانداژفشاری چهارلایه،ورزش وتوصیه های مربوط به مراقبت های پوستی)است را دریافت میکنند. این فاز به مدت 3 هفته،5 جلسه در هفته و هر جلسه به مدت 60 دقیقه اجرا می شود .<br>بیمارانی که در گروه مداخله قرار دارند قبل از برنامه CDT تکنیک های تن آرامی را که شامل آرام سازی عضلانی پیشرونده است انجام می دهند. تکنیک های تن آرامی بدین صورت انجام می شود فرد گروههای عضلانی مختلف را به مدت 10-5 ثانیه منقبض کرده و سپس به حالت استراحت بر می گر داند. انقباض ابتدا از عضلات سرو صورت شروع وبه عضلات ساق و انگشتان پا ختم می شود.<br>این تکنیک ها به مدت 30 دقیقه درهفته اول ،در ابتدای جلسه توسط درمانگر و در 2 هفته باقی مانده توسط خود فرد و با نظارت درمانگر قبل از درمان CDT در مراکز نام برده ادامه دارد.<br>پس از خاتمه فاز اول درمان ارزیابی مجدد که شامل حجم ادم لنفاوی و تکمیل تست HADS است از شرکت کننده ها صورت می گیرد و افراد وارد فاز دوم درمان می شوند.<br>فاز دوم به مدت 6-4 هفته است؛ پس از اتمام فاز اول از افراد خواسته می شود که 6-4 هفته بعد به کلینیک مراجعه کنند. افرادی که در هفته 4 مراجعه نکنند با آن ها تماس گرفته می شود و از این افراد خواسته می شود جهت پیگیری مراجعه کنند.<br>فاز دوم مداخله نیز با ارائه آموزش های لازم به بیمار در منزل توسط وی انجام خواهد شد. ر طی این فاز بیمار هرروز به مدت 60 دقیقه برنامه CDT شامل ماساژ تخلیه لنف توسط خود بیمار، استفاده از آستین فشاری در روز و بانداژکم کش چند لایه در شب، انجام ورزش با بانداژ و رعایت مراقبتهای پوستی را انجام می دهد و در گروه مداخله، بیماران 30 ددقیقه قبل از انجام CDT، تکنیک های تن آرامی را انجام می دهند.<br>برای پیگیری انجام این تکنیک ها 2 هفته یکبار با این افراد تماس گرفته می شود.<br>در پایان این فاز برای بار سوم از افراد ارزیابی به عمل می آید.	
پس از انجام مراحل کسب اجازه تحقیق ،به محل تحقیق مراجعه ودر مورد اهداف و نحوه انجام پژوهش به مسئولین توضیح داده و موافقت کسب خواهد شد. سپس تحقیق خود را به افراد واجد شرایط پژوهش معرفی کرده و به آن ها توضیحات لازم در خصوص هدف و نحوه اجرا ی پژوهش داده می شود. افراد مورد پژوهش کاملا در جریان تحقیقاتی بودن کار، فرآیند و اهداف پژوهش قرار گرفته و	**ملاحظات و مشکلات اخلاقی طرح**

	پس از توجیه، از آن ها رضایت نامه کتبی در مورد حضور در پژوهش کسب خواهد شد. همچنین به افراد مورد پژوهش اطمینان داده خواهد شد که اطلاعات شخصی شرکت کنندگان و اطلاعات گرد آوری شده به صورت محرمانه محفوظ خواهد ماند و بدین منظور در فرم پرسشنامه به جای ذکر نام و نام خانوادگی به آنان کد داده خواهد شد. کلیه افراد مورد پژوهش برای شرکت در تحقیق آزاد بوده و جهت ادامه شرکت در پژوهش هیچ اجباری وجود ندارد. در صورت بروز هر گونه عوارض ناشی از درمان ،در جهت رفع آن اقدام خواهد شد.
**راه حل مشکلات اخلاقی**	گرفتن رضایت نامه جهت شرکت در تحقیق ازاد بودن افراد برای ترک کردن تحقیق . به دلیل اینکه در این تحقیق مداخله مورد نظر فقط در گروه مورد مطالعه اجرا می شود به افراد شرکت کننده در گروه کنترل اطمینان داده می شود در صورت مؤثر بودن مداخله برای ان ها نیز انجام می شود
**آیا طرح رضایتنامه کتبی آگاهانه از آزمودنی دارد**	بله
