# Supplementary material for: The effect of relaxation techniques on edema, anxiety and depression in post-mastectomy lymphedema patients undergoing comprehensive decongestive therapy: A clinical trial
Source: PLoS One. 2018 Jan 5;13(1):e0190231. doi: 10.1371/journal.pone.0190231 (PMC5755759; doi:10.1371/journal.pone.0190231)
Supplement: S2 Appendix — (PDF) [file pone.0190231.s002.pdf]

Study Protocol

**Title**	The effect of relaxation techniques on lymph edema volume ,anxiety and depression in women with lymphedema under treatment by Complex Decongestive Therapy
**Key words**	Relaxation techniques, Complex Decongestive Therapy (CDT), Lymphedema, Anxiety, Depression
**Presenter**	School of Rehabilitation, Shahid Beheshti University of Medical Sciences
**Type of Study**	Clinical Trial
**Objective**	Evaluating the effect of relaxation techniques on lymph edema volume , anxiety and depression in breast cancer patients with lymphedema under treatment by complex decongestive therapy in start of study compared to months 3 and 9
**Study Population**	In this study, the research community are patients with breast cancer induced lymphedema of the upper limb (other than mild) with HADS score of 8 and above.<br>Target population: All women with unilateral breast cancer that lead to breast removal (mastectomy) and passed primary adjuvant treatments.<br>Study population: patients with lymphedema due to breast cancer treatment who are referred to lymphedema Clinic of Seyed Khandan Center
**Sampling & Sample size**	In this study non-probability sampling will be applied. During the study, the researcher will be present at the Center and all referred patients with lymphedema and inclusion criteria will be recruited in the study.<br>Sample size was computed be Annette Loudon (2014) study which was in this field. Effect mean difference was considered 0.17, standard deviation equal to 0.15, α and β equal to 0.05 and 0.1 respectively.<br>The number of samples was calculated 32 patients, 16 people in the control group and 16 patients in intervention group.
**Materials& Methods**	Research is started after the official presentation of project to authorities of study place and their approval. Patients with unilateral lymphedema due to breast cancer treatment who have gained inclusion criteria for the

	study are enrolled and fulfill a check list about demographic and clinical information.<br>Definitely in the study plan, randomization in groups is not possible. During 2-3 weeks of treatment of the two groups, they will certainly come in contact with each other and there is possibility of relaxation and its impact in the control group.(Control Contamination)<br>So, At the beginning, patients are treated by CDT alone, after the completion of the first group sample size, relaxation will be added to the CDT.<br>To adjust the effects of time and treatment on outcome, we will try to select the second group in similar time horizons and treatment protocol. If needed, They will be matched for potential confounders such as the volume of edema and age. Even though, it is not a randomized trial, we try to make them more comparable.<br>We do CDT and relaxation treatment after completing the sample size of CDT alone group. Because it is probable that therapists' familiarity with the techniques of relaxation may have effect on subsequent treatments. She may apply some parts of relaxation on patients during CDT.<br>Assessments conducted before and after the intervention consist of measuring volume of lymphedema using Water displacement method, measuring anxiety and depression by Hospital Anxiety and Depression Scale questionnaire (HADS). For preventing information bias, measurements are done by someone other than the main researchers.
**Method of Investigation**	This research consists of two phases:<br>At start of phase one, outcome assessments are made. The first phase lasts 3 weeks. Patients are treated 5 days a week and each session lasts 60 minutes.<br>At the first phase, both groups receive routine CDT consisting of Manual lymph drainage, Multilayers bandaging, rehabilitation exercise and advice about the care of the skin.<br>Patients in the intervention group receive relaxation techniques before CDT program. It includes progressive muscle relaxation. Relaxation aims different muscle groups. Each group of muscles are contracted for 5-10 seconds and then they are relaxed. Relaxation techniques start from head and face muscles and end at legs and toes muscles. This technique lasts 30 minutes at the beginning of the sessions. At first week, it is

	trained by the therapist and in the remaining two weeks, it is achieved by patient herself and supervision of therapist. After relaxation, CDT continues.<br>At the end of the first phase of lymphedema treatment, re-evaluation of outcomes are achieved which include edema volumetry and fulfilling HADS questionnaire.<br>The second phase is a period of 4-6 weeks, after completing the first phase of treatment. They should attend the clinic 4-6 weeks later for follow up visit. If patients don't attend the clinic after 4 weeks, researcher will call them and invite them for check-up.<br>At the second phase of intervention, necessary training are provided for patients. Patients should do daily CDT at home for 60 minutes. In maintenance phase it consists of manual lymph drainage, using compression arm-sleeve at day, using multilayer bandage at nights, rehabilitation exercise and skin care. In intervention group, patients do relaxation techniques for 30 minutes before CDT.<br>Achieving treatments in maintenance phase are checked by researchers by telephone call every two weeks.<br>At the end of this phase, Outcomes are re-evaluated for the third time.
**Ethical Considerations**	After issuing the research permission, researchers present the project to the official authorities of study place and obtain their approval. Researchers explain the objectives and methodology of study to them. Then they introduce their research to eligible patients. Their explanations are about the objectives and implementation of research.<br>All patients who accept to participate in study, sign an informed consent.<br>The research will also ensure that the personal information of participants will remain confidential Reserved. For this purpose, a code will be given to them instead of using their name in questionnaire.<br>All subjects were free to participate in the study and there is no obligation to continue their participation during research time.<br>Researches are responsible for any side effect of treatment during study and will solve it.<br>Because relaxation is done only in intervention group, participants in control group are assured that they will receive that technique later, it will be effective.

**Is there any written informed consent**	Yes
