# Supplementary material for: The effect of relaxation techniques on edema, anxiety and depression in post-mastectomy lymphedema patients undergoing comprehensive decongestive therapy: A clinical trial
Source: PLoS One. 2018 Jan 5;13(1):e0190231. doi: 10.1371/journal.pone.0190231 (PMC5755759; doi:10.1371/journal.pone.0190231)
Supplement: S1 Text — (PDF) [file pone.0190231.s003.pdf]

مقاله پژوهشی

فصلنامهٔ بیماری‌های پستان ایران، سال هشتم، شماره اول، بهار ۱۳۹۴

تأثیر تکنیک‌های تن‌آرامی بر میزان حجم ادم لنفاوی، اضطراب و افسردگی در زنان دارای ادم لنفاوی تحت درمان با احتقان‌زدایی ترکیبی

نوید میرزاخانی: دانشکده توانبخشی، دانشگاه علوم پزشکی شهید بهشتی، تهران، ایران

بهاره عباسی: دانشکده توانبخشی، دانشگاه علوم پزشکی شهید بهشتی، تهران، ایران

شهپر حقیقت*: گروه پژوهشی کیفیت زندگی مبتلایان به سرطان، مرکز تحقیقات سرطان پستان، جهاد دانشگاهی، تهران، ایران

لیلا انگوتی: بیمارستان شهدای تجریش، دانشگاه علوم پزشکی شهید بهشتی، تهران، ایران

اشکان ایرانی: دانشکده توانبخشی، دانشگاه علوم پزشکی شهید بهشتی، تهران، ایران

سید مهدی طباطبایی: دانشکده توانبخشی، دانشگاه علوم پزشکی شهید بهشتی، تهران، ایران

چکیده

مقدمه: ادم لنفاوی اندام فوقانی یکی از علل مهم ناراحتی روانی و جسمی در بیماران سرطان پستان محسوب می‌شود. سطح بالایی از اضطراب و افسردگی در بیماران ادم لنفاوی اتفاق می‌افتد. این مطالعه با هدف بررسی تأثیر تکنیک‌های تن‌آرامی بر میزان حجم ادم لنفاوی، اضطراب و افسردگی در زنان دارای ادم لنفاوی تحت درمان با احتقان‌زدایی ترکیبی (CDT) بود.

روش بررسی: پژوهش حاضر که کار آزمایی بالینی نیمه تجربی است، ۳۱ زن مبتلا به ادم لنفاوی (اختلاف حجم دو اندام بیش از ۲۰۰ سی‌سی) در دو گروه کنترل (۱۶ نفر) و آزمون (۱۵ نفر) قرار گرفتند. ابزار جمع‌آوری داده‌ها شامل؛ اندازه‌گیری حجم ادم لنفاوی از طریق تانک حجم‌سنج، میزان اضطراب و افسردگی با استفاده از پرسشنامه HADS قبل، پایان فاز اول و ۶-۴ هفته پس از اتمام فاز اول درمان بود. مداخله در دو فاز انجام شد. فاز اول به مدت ۳ هفته (۶ روز در هفته) که در گروه کنترل CDT ودر گروه آزمون قبل از انجام درمان CDT تکنیک‌های تن‌آرامی شامل آرام‌سازی عضلانی پیش‌رونده توسط محقق در کلینیک انجام شد و فاز دوم به مدت ۶-۴ هفته در منزل توسط بیمار در هر دو گروه ادامه پیدا کرد.

یافته‌ها: یافته‌های مطالعه نشان داد که میزان حجم ادم، نمرات اضطراب و افسردگی در گروه آزمون به ترتیب ۶۳/۶، ۵۴/۱ و ۶۵/۵ درصد و در گروه کنترل به ترتیب ۶۰/۷، ۳۱/۴ و ۳۵/۲ درصد کاهش یافتند. اختلاف معنی‌داری در درصد کاهش نمره افسردگی در طی فاز اول ($p=0.02$)، کل دوره پژوهش ($p=0.004$) و درصد کاهش نمره اضطراب در کل دوره پژوهش ($p=0.005$) بین دو گروه مشاهده شد.

نتیجه‌گیری: انجام تکنیک‌های تن‌آرامی موجب کاهش نمره اضطراب و افسردگی و نیز حجم ادم در بیماران دارای ادم لنفاوی می‌گردد.

واژه‌های کلیدی: تکنیک‌های تن‌آرامی، ادم لنفاوی، اضطراب، افسردگی، احتقان‌زدایی ترکیبی.

* نشانی نویسنده پاسخگو: تهران، میدان ونک، بزرگراه حقانی، ابتدای خیابان گاندی جنوبی، شماره ۱۴۶، شهپر حقیقت.

نشانی الکترونیک: sha_haghighat@yahoo.com

مقدمه

سرطان پستان در سراسر جهان شایع‌ترین سرطان در زنان است (۱). مطابق گزارش ثبت سرطان ایران در سال ۱۳۸۸ تعداد ۷۵۸۲ مورد سرطان پستان در زنان ایرانی شناسایی شد (۲) سن ابتلا به سرطان پستان ۵ تا ۱۰ سال کمتر از کشورهای دیگر است (۱). مطالعات نشان دادند که سرطان پستان، از همان ابتدای تشخیص می‌تواند باعث ترس زیاد، ناامیدی و آسیب روانی گردیده و همه ابعاد زندگی فرد شامل جسمی، روانی، اجتماعی و معنوی را به چالش بکشاند (۳) در مطالعه‌ای که توسط مالاموس در سال ۲۰۱۰ با هدف بررسی افسردگی و اضطراب در زنان مبتلا به سرطان پستان انجام گرفت، نشان داد میزان افسردگی و اضطراب به طور قابل توجهی در گروه بیمار در مقایسه با افراد سالم بالاتر بود (۴).

امروزه با استفاده از روش‌های غربالگری و تشخیص زودرس این بیماری و همچنین پیشرفت متدهای درمانی تحول بزرگی در درمان این بیماری ایجاد شده است (۵). همان‌طور که زنان زندگی طولانی‌تری پس از تشخیص سرطان پستان دارند اثرات جانبی درمان سرطان پستان مثل ادم لنفاوی افزایش می‌یابد (۶) ادم لنفاوی یکی از عوارض اصلی سرطان پستان و درمان آن است و می‌تواند عوارض طولانی مدت جسمی و روانی در بیماران داشته باشد (۷). گزارش‌ها از شیوع ادم لنفاوی خیلی وسیع است و دامنه‌ای از ۸/۴٪ تا ۲۱/۴٪ دارد (۸). ادم لنفاوی یک وضعیت مزمن پزشکی است که بر مناطق عملکرد کاری شامل فعالیت‌های روزمره زندگی، فعالیت‌های اجتماعی و ارتباطات بین فردی، شغل و فعالیت‌های مربوط به خانه مراجع تأثیر می‌گذارد (۹). مسایل روانی اجتماعی قابل توجه که همراه با ادم لنفاوی در سرطان پستان وجود دارد شامل: کاهش اطمینان به بدن، عدم فعالیت جسمی، آشفتگی‌های روانی از قبیل عصبانیت، غم، علایم افسردگی، ضعف در تصویر بدنی، مشکلات جنسی، اضطراب و اجتناب از حضور در جامعه است (۱۰). از عوارض سایکولوژیک لنف‌ادم افسردگی و انزوای فرد از اجتماع است که کارایی و توانایی بیمار در محیط کار و خانه را کاهش می‌دهد (۱۱).

احتقان‌زدایی ترکیبی یک تکنیک غیرجراحی مؤثر برای درمان ادم لنفاوی است که به وسیله انجمن لنفولوژی توصیه شده است (۱۲). نوعی درمان توانبخشی شامل ماساژ MLD[1]، بانداژ چند لایه‌ای پرفشار، ورزش و مراقبت از پوست است (۱۳). آرام‌سازی یا تن آرامی از روش های کاهش تنش است (۱۷). در این روند فرد با انجام حرکات انقباضی و برگشت آنها به طور ارادی به حالت شل و منبسط طی ۵ تا ۱۰ ثانیه سبب افزایش جریان خون می‌شود در انتهای انجام آن فعالیت، فرد درجاتی از کاهش اضطراب یا نگرانی را احساس می‌کند (۱۴).

مکلور با انجام تن‌آرامی و تمرین درمانی بر روی زنان دارای ادم لنفاوی به این نتیجه رسید که انعطاف‌پذیری بازو،کیفیت زندگی و خلق در طی ۳ ماه پیگیری بهبود می‌یابد (۱۵). لوداندر یک مطالعه به بررسی تأثیر یوگا بر حجم ادم لنفاوی در زنان دارای سرطان پستان پرداخت و به این نتیجه رسید که یوگا اثر مثبت در کاهش حجم ادم و علایم همراه آن دارد (۱۶). قربانی تحقیقی روی زنان مبتلا به سرطان پستان جهت تعیین تأثیر دو روش یوگا و پیلاتس بر دامنه حرکتی، ادم و درد اندام فوقانی انجام دادکه نتایج افزایش دامنه حرکتی، کاهش ادم و درد را نشان داد (۱۷).

مطالعات مختلف نشان داده‌اند که مسایل روانی در یک کلینیک ادم لنفاوی چند بعدی است که این مسایل نیاز به مداخله دارند (۸) برنامه کاهش حجم به تنهایی برای بهبود قابل توجه عملکرد ضعیف این افراد در بعد روان‌شناختی، مؤثر نیست (۱۸). محققان زیادی همواره بر به‌کارگیری روش‌های غیردارویی در کاهش عوارض سرطان پستان اهتمام ورزیده‌اند و تن‌آرامی را روشی اثربخش در کاهش آشفتگی روانی ناشی از سرطان ذکر کردند (۱۹). با توجه شیوع بالای سرطان پستان در ایران و نیز ادم لنفاوی ناشی از درمان آن که موجب ناتوانایی‌های متعدد جسمی و روانی در این افراد می‌شود، این پژوهش به بررسی تأثیر تکنیک‌های تن‌آرامی بر میزان حجم ادم لنفاوی، اضطراب و افسردگی در زنان دارای ادم لنفاوی تحت درمان با احتقان‌زدایی ترکیبی پرداخته است. در صورت هم‌افزایی تاثیر آرام‌سازی با روش احتقان‌زدایی ترکیبی در بهبود علایم جسمانی و روانی این بیماران، می‌توان به راهکارهای کاربردی جهت کنترل چند بعدی و موثرتر این عارضه نایل آییم.

[1]Manual Lymphatic Drainage

مواد و روش‌ها

مطالعه حاضر، مطالعه‌ای نیمه‌تجربی با گروه کنترل و آزمون است. در این طرح تصادفی کردن دو گروه امکان پذیر نبود، زیرا در طی ۳ هفته اجرای فاز اول درمان دو گروه قطعا با هم تماس پیدا می‌کردند و احتمال انجام تن‌آرامی و تأثیر آن در گروه کنترل هم وجود داشت. لذا جهت افزایش توان مطالعه، ابتدا در یک مقطع زمانی ۶ ماهه بیماران تحت CDT تنها قرار گرفتند و بعد از تکمیل حجم نمونه گروه اول و تکمیل پرسشنامه‌های لازم، درمان تن‌آرامی به CDT اضافه شد، که بر روی زنان مبتلا به ادم لنفاوی ناشی از سرطان پستان، مراجعه‌کننده به کلینیک فیزیوتراپی سیدخندان در سال ۱۳۹۲ که دارای معیارهای زیر بوده‌اند، انجام شده است.

علی‌رغم عدم تصادفی‌سازی در ابتدای مطالعه، این نحوه ورود بیماران موجب شد که پژوهشگر دخالتی در تخصیص افراد به گروه‌های تحت مطالعه نداشته باشد. ضمن اینکه در بازه زمانی مذکور تغییری در استانداردهای درمانی کلینیک نیز ایجاد نشده بود.

تمام زنان مبتلابه سرطان پستان یک طرفه و تحت عمل جراحی برداشت کامل یا جزئی پستان قرار گرفته باشند، دارای ادم لنفاوی یک طرفه در ناحیه اندام فوقانی باشند (اختلاف حجم دو اندام بیش از۲۰۰ سی‌سی)، دارای حداقل نمره ۸ در تست HADS[2] در هر یک از آیتم‌های اضطراب و افسردگی، عدم دریافت درمان ادم لنفاوی و یا تکنیک‌های تن‌آرامی قبل از مطالعه، نداشتن بیماری شدید روانی، اتمام دوره پرتودرمانی و شیمی‌درمانی.

از آنجا که انجام تکنیک‌های تن‌آرامی در محیط پژوهش در گروه کنترل امکان پذیر بود، ابتدا نمونه‌گیری این گروه و سپس نمونه‌گیری گروه آزمون صورت گرفت و حجم نمونه با توجه به مطالعات قبلی و احتساب (α=۰/۰۵) و (β=۰/۱)، ۱۶نفر برای هر گروه محاسبه شد بدین ترتیب با روش نمونه‌گیری در دسترس از ۴۹ بیمار مراجعه‌کننده طی انجام مطالعه به تدریج به دلیل عدم تکمیل درمان یا نیاز به سایر درمان‌های تکمیلی سرطان پستان، ۱۸ نفر از مطالعه خارج شدند و نهایتاً ۳۱ نفر باقی ماندند.که به ترتیب ۱۶ نفر در گروه کنترل و ۱۵ نفر در گروه آزمون قرار گرفتند.

در این پژوهش از پرسشنامه بررسی اطلاعات دموگرافیک جهت ارزیابی اطلاعات دموگرافیک بیماران، تانک حجم‌سنج برای اندازه‌گیری میزان حجم ادم با روش جابجایی آب، با معلق کردن ابتدا اندام سالم و سپس اندام ادم دار تا ۲ سانتی‌متر پایین‌تر از چین زیر بغل در تانک محتوی آب انجام شد (۲۰) و پرسشنامه اضطراب و افسردگی بیمارستانی (HADS) که شامل ۱۴ آیتم است که ۷ آیتم آن مربوط به اضطراب و ۷ آیتم دیگر مربوط به افسردگی است. هر آیتم پرسشنامه بین ۳-۰ نمره‌دهی می‌شود. در این پژوهش به منظور بررسی تعیین میزان اضطراب و افسردگی بیمار مبتلا به سرطان پستان از برگردان ایرانی پرسشنامه اضطراب و افسردگی بیمارستانی (HADS) که روایی و پایایی آن در پژوهشکده علوم بهداشتی جهاددانشگاهی توسط آقای دکتر منتظری و همکارانش در سال ۲۰۰۳ با الفای کرونباخ ۰/۷۸ برای آیتم‌های مربوط به اضطراب و ۰/۸۶ برای آیتم‌های افسردگی، استفاده شد (۲۱).

پس از گرفتن شرح حال و تکمیل پرسشنامه‌ها و اندازه-گیری میزان حجم ادم لنفاوی، دو فاز درمانی بدین صورت که فاز اول یا همان فاز درمانی، در ۳ هفته متوالی (۶ روز در هفته به جز جمعه‌ها) و هر جلسه به مدت ۶۰ دقیقه در گروه کنترل با درمان احتقان‌زدایی ترکیبی که شامل؛ انجام ماساژ MLD، بانداژ چند لایه‌ای، آموزش نحوه انجام یکسری ورزش‌های دامنه حرکتی به همراه بانداژ یا پوشش فشاری و آموزش مراقبت از پوست و ناخن و در انتهای این فاز، بیمار مورد ارزیابی قرار می‌گرفت. آموزش‌های لازم در این فاز به افراد جهت ادامه درمان در فاز دوم پژوهش داده شد. در فاز دوم، با ارایه بروشور و سی‌دی آموزشی مربوط به جلسات آموزشی درمان ادم لنفاوی اجرا شده به بیمار، دررمان در منزل توسط وی انجام شدکه شامل؛ انجام ماساژ SLD[3] توسط خود بیمار، استفاده از دستکش فشاری نیز انجام بانداژ چند ی در شب، انجام ورزش‌ها با پوشش آستین فشاری، ادامه مراقبت از پوست و ناخن.

در گروه آزمون قبل از انجام CDT تکنیک‌های تن‌آرامی که شامل آرام‌سازی عضلانی پیشرونده بود که فرد باید با توجه به دستور درمانگر گروه‌های عضلانی مورد نظر را به مدت ۷-۵ ثانیه منقبض و به مدت ۱۰ ثانیه شل می‌کرد، انجام شد. فاز دوم نیز هر روز به همین صورت در منزل

[2] Hospital Anxiety and Depression Score

[3] Self-Lymphatic Drainage

توسط خود بیمار با انجام این تکنیک‌ها قبل از درمان CDT ادامه پیدا کرد. ارزیابی‌ها در انتهای فاز اول درمان و فاز دوم (۶-۴ هفته) مجددا صورت گرفت. در دوره پیگیری تماس تلفنی با افراد شرکت‌کننده در پژوهش جهت کنترل درمان و پاسخ به سوالات، توسط محقق انجام شد.

پس از جمع‌آوری داده‌های قبل، پایان فاز اول و پیگیری از پرسشنامه‌ها و کدبندی اطلاعات پژوهش از نرم‌افزار SPSS نسخه ۱۹، جهت تجزیه و تحلیل داده‌ها استفاده شد. تفاضل مقدار پیامد در انتهای فاز اول از شروع درمان نسبت به مقدار اولیه بعنوان تغییرات فاز اول در نظر گرفته شد. در تعریف فاز پیگیری، شروع و خاتمه پیامد به ترتیب انتهای فاز اول و انتهای پیگیری منظور شد و در تعریف تغییرات کل دوره، شروع و خاتمه تغییرات از شروع مطالعه تا خاتمه زمان پیگیری محاسبه شد. در مقایسه پیامدهای پژوهش، با توجه به نرمال بودن توزیع متغیر ازآزمون تی استودنت برای نمونه‌های مستقلو آنالیز اندازه‌گیری مکرر برای بررسی تغییرات داده‌ها در طول زمان استفاده شد. سطح معنی‌داری خطای آلفا در کلیه آزمون‌ها ۰/۰۵ در نظر گرفته شد.

یافته‌ها

مشخصات دموگرافیک و بالینی افراد تحت مطالعه به تفکیک گروه‌های آزمون و کنترل در جداول ۱ و ۲ ارائه گردیده است.

همان طور که نتایج نشان می‌دهد؛ میانگین سنی زنان در گروه کنترل و آزمون ۵۲/۰۶ و ۵۵/۴ سال، میانگین شاخص توده بدنی در گروه کنترل ۲۹/۰۳ کیلوگرم بر متر مربع و در گروه آزمون ۳۰/۱۴ کیلوگرم بر متر مربع، میانگین فاصله زمان جراحی تا بروز لنف‌ادم در دو گروه به ترتیب ۳۲/۹۳ و ۴۲/۴ ماه بود. میانگین تعداد غدد لنفاوی خارج شده در گروه کنترل ۱۳ عدد و در گروه آزمون ۱۲ عدد، میانگین تعداد غدد لنفاوی درگیر در دو گروه به ترتیب ۵ و ۴ عدد می‌باشد، همچنین بر اساس جدول ۲، اغلب بیماران در گروه کنترل (۱۰۰٪) و آزمون (۸۶/۶٪) متأهل بودند، از نظر سطح تحصیلات در گروه کنترل ۵۶/۲٪ دارای مدرک دیپلم و درگروه آزمون ۴۰٪ افراد دارای مدرک زیر دیپلم بودند. جراحی ماستکتومی کامل رایج‌ترین نوع جراحی در دو گروه کنترل و آزمون بوده است (۸۱/۲٪ در برابر ۸۶/۶٪). سابقه پرتو درمانی در ۸۱/۲٪ افراد گروه کنترل و ۸۰٪ گروه آزمون و شیمی‌درمانی در دو گروه به ترتیب ۹۲/۷٪ و ۱۰۰٪ افراد وجود داشته است. مقایسه مشخصات دموگرافیک و بالینی دو گروه با استفاده از آزمون‌های تی استودنت (جدول ۱) و کای دو (جدول ۲) در سطح خطای ۰/۰۵ اختلاف معنی داری بین دو گروه نشان نداد.

بررسی نتایج قبل از مداخلات نشان داد که دو گروه از نظر میزان حجم ادم لنفاوی، اضطراب و افسردگی تفاوت معنی‌داری نداشتند.

جدول ۱: مقایسه میانگین و انحراف معیار مشخصات جمعیتی و بالینی گروه‌های تحت مطالعه (n=31)

متغیر	گروه کنترل (n=16)		گروه آزمون (n=15)		p-value
	میانگین (±انحراف معیار)	دامنه	میانگین (±انحراف معیار)	دامنه	
سن (سال)	۵۲/۰۶ (±۱۰/۱۸)	۳۷-۷۵	۵۵/۴۷ (±۱۱/۵۱)	۳۷-۷۴	۰/۴
شاخص توده بدنی (کیلوگرم بر متر مربع)	۲۹/۰۳ (±۴/۳۱)	-۲۱/۸۳ ۳۷/۶۵	۳۰/۱۴(±۴/۴۷)	۲۴/۴۶-۳۹/۱۴	۰/۴۸
طول مدت ادم (ماه)	۸/۵۶(±۷/۸۹)	۱-۲۹	۱۰/۷۶(±۱۴/۰۸)	۰/۵-۴۸	۰/۵۹
فاصله جراحی تا بروز لنف ادم (ماه)	۳۲/۹۳(±۲۵/۶۳)	۴-۹۶	۴۲/۴(±۳۴/۹۹)	۸-۹۵	۰/۳۹
تعداد غدد لنفاوی خارج شده	۱۳/۶۲(±۶/۹۱)	۱-۲۵	۱۲/۱۳(±۴/۵۱)	۴-۲۱	۰/۴۸
تعدادغدد لنفاوی درگیر	۵/۱۸(±۵/۱۶)	۰-۱۹	۴/۲(±۳/۷۲)	۰-۱۲	۰/۵۴

جدول ۲: مقایسه فراوانی مطلق و نسبی مشخصات جمعیتی و بالینی گروه‌های آزمون و کنترل (n = 31)

متغیر	گروه کنترل (n=16)		گروه آزمون (n=15)		p-value
	تعداد	درصد	تعداد	درصد	
**وضعیت تأهل**					۱
مجرد	۰	۰	۱	۶/۷	
متأهل	۱۶	۱۰۰	۱۳	۸۶/۶	
بیوه/مطلقه	۰	۰	۱	۶/۷	
**تحصیلات**					۰/۹۵
بی‌سواد	۱	۷/۳	۱	۶/۶	
زیر دیپلم	۴	۲۵	۶	۴۰	
دیپلم	۹	۵۶/۲	۴	۲۶/۷	
دانشگاهی	۲	۱۲/۵	۴	۲۶/۷	
**دست غالب**					۰/۰۸
راست	۸	۵۰	۱۲	۸۰	
چپ	۸	۵۰	۳	۲۰	
**اندام مبتلا**					۰/۳۵
راست	۸	۵۰	۵	۳۳/۴	
چپ	۸	۵۰	۱۰	۶۶/۶	
**نوع جراحی**					۰/۶۸
برداشت کامل پستان	۱۳	۸۱/۲	۱۳	۸۶/۸	
حفظ پستان	۳	۱۸/۸	۲	۱۳/۲	
**پرتودرمانی**					۰/۹۳
دارد	۱۳	۸۱/۲	۱۲	۸۰	
ندارد	۳	۱۸/۸	۳	۲۰	
**شیمی‌درمانی**					۰/۳۳
دارد	۱۵	۹۲/۷	۱۵	۱۰۰	
ندارد	۱	۷/۳	۰	۰	

با استفاده از آزمون تی استودنت، میانگین تغییرات پیامدهای حجم ادم، اضطراب و افسردگی در مقاطع مختلف زمانی مورد مقایسه قرار گرفت. میانگین درصد کاهش حجم ادم طی فاز اول مداخله در گروه کنترل ۴۵/۵ و آزمون ۴۶/۸۴٪ و در دوره پیگیری در گروه کنترل ۲۸/۱۴٪ و در گروه آزمون ۳۰/۳۴٪ بود.

همان‌طور که جدول ۳ نشان می‌دهد، درصد کاهش حجم در کل دوره در گروه آزمون نسبت به گروه کنترل کمی بیشتر بود (۶۳/۵۹٪ در برابر ۷۲/۰٪). لیکن مقایسه میانگین‌ها با استفاده از آزمون تی مستقل، تفاوت معنی‌داری را بین دو گروه در هیچ‌یک از مراحل درمانی نشان نداد. میانگین درصد کاهش نمره اضطراب در فاز اول، پیگیری و کل دوره در گروه کنترل به ترتیب ۲۵/۲٪ ۹/۶٪ و ۳۱/۳٪ و در گروه آزمون ۴۲/۸٪، ۱۵/۸٪ و ۵۴/۱٪ بود که در کل دوره (p=0.005) تفاوت معنی‌داری در سطح خطای ۵٪ بین دو گروه مشاهده شد. میانگین درصد کاهش نمره افسردگی بین دو گروه در فاز اول (p=0.02) و کل دوره پژوهش (p=0.004) اختلاف معنی‌داری نشان داد (جدول ۳).

تغییرات میانگین حجم ادم، نمره اضطراب و افسردگی در دو گروه در طی بازه زمانی شروع مطالعه تا پایان دوره پیگیری در جدول ۴ خلاصه شده است. میانگین حجم ادم و نمرات اضطراب و افسردگی در دو گروه در طول زمان روند کاهشی را نشان می‌دادند. در آنالیز داده‌های مکرر، رابطه معنی‌داری در تغییرات میانگین نمرات افسردگی (p=0.007) بین دو گروه مشاهده شد که در نمودار ۱ نمایش داده شده است.

جدول ۳: میانگین و انحراف معیار درصد کاهش حجم ادم، اضطراب و افسردگی در دو گروه مورد پژوهش در فاز اول، پیگیری و کل دوره درمان

متغیر	زمان	گروه کنترل(n=۱۶)		گروه آزمون(n=۱۵)		p-value
		میانگین	انحراف معیار	میانگین	انحراف معیار	
**حجم**	فاز اول	۴۵/۵۹	۱۲/۳۹	۴۶/۸۴	۱۵/۶۵	۰/۸
	پیگیری	۲۸/۱۴	۲۰/۴۵	۳۰/۳۴	۱۷/۲۹	۰/۷۴
	ابتدا تا انتها	۶۰/۷۲	۱۵/۱۲	۶۳/۵۹	۱۱/۳۵	۰/۵۵
**اضطراب**	فاز اول	۲۵/۲۸	۳۴/۳۹	۴۲/۸۸	۱۷/۹۲	۰/۰۸
	پیگیری	۹/۶۷	۱۹/۱	۱۵/۸۳	۲۱/۷۹	۰/۴۱
	ابتدا تا انتها	۳۱/۳۷	۲۶/۲	۵۴/۱۲	۱۳/۵۸	۰/۰۰۵
**افسردگی**	فاز اول	۲۵/۲۲	۴۶/۳	۵۶/۹۳	۲۵/۰۶	۰/۰۲
	پیگیری	۵/۱۶	۳۸/۹۷	۲۹/۶۴	۲۴/۳۹	۰/۰۵۵
	ابتدا تا انتها	۳۵/۰۸	۳۸/۷۲	۶۹/۴۵	۱۸/۸۵	۰/۰۰۴

جدول ۴: بررسی تغییرات میانگین میزان حجم ادم، و نمرات اضطراب و افسردگی در دو گروه در طی سه نوبت اندازه‌گیری قبل از درمان، انتهای فاز اول و انتهای پیگیری

متغیر	مراحل اندازه‌گیری	گروه آزمون (انحراف معیار±) میانگین	گروه کنترل (انحراف معیار±) میانگین	p-value
**حجم ادم**				۰/۷۴
	قبل از درمان	۸۸۴/۳۲ (± ۶۹۳/۹۶)	۹۶۴/۵۹ (±۴۴۶/۶۱)	
	خاتمه فاز اول	۴۶۳/۰۳ (± ۳۴۰/۴۴)	۵۰۵/۱۸ (± ۲۵۰/۱۲)	
	خاتمه پیگیری	۳۴۳/۰۷ (± ۳۰۵/۶۶)	۳۵۴/۶۳ (± ۲۰۲/۷)	
**نمره اضطراب**				۰/۱۸
	قبل از درمان	۱۱/۴ (± ۲/۴)	۱۱/۷ (± ۲/۸)	
	خاتمه فاز اول	۸/۳ (±۳/۷)	۶/۷ (± ۲/۵)	
	خاتمه پیگیری	۷/۷ (± ۳/۳)	۵/۴ (± ۱/۹)	
**نمره افسردگی**				۰/۰۰۷
	قبل از درمان	۱۰/۱ (± ۲/۲)	۹/۶ (± ۱/۴)	
	خاتمه فاز اول	۷/۵ (± ۴/۵)	۴/۲ (± ۲/۵)	
	خاتمه پیگیری	۶/۴ (± ۳/۶)	۲/۹ (±۱/۷)	

*سطح معنی‌داری ۰/۰۵ در نظر گرفته شده است.

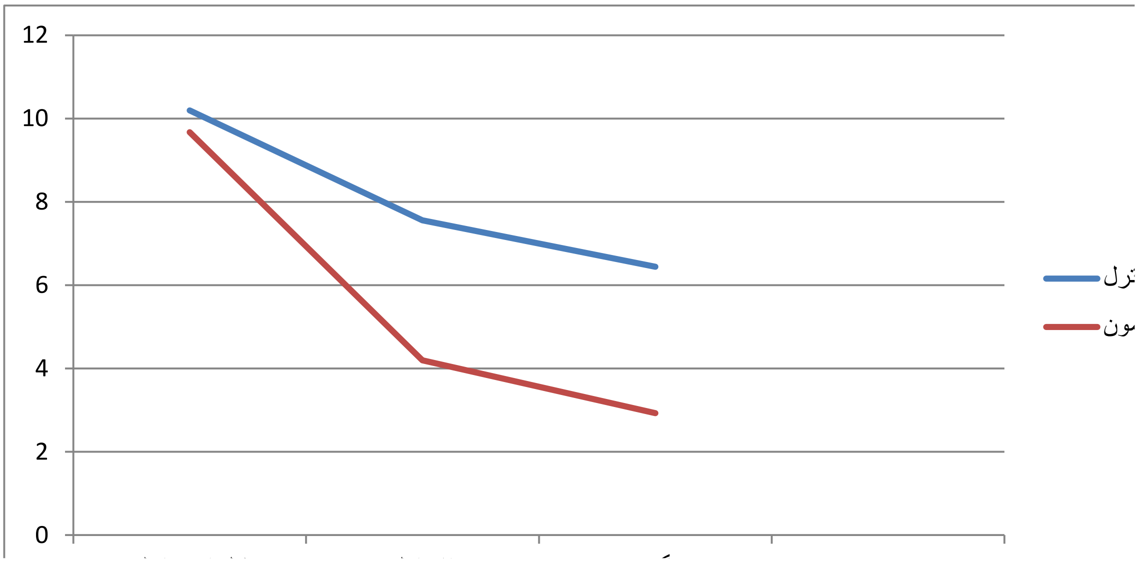

نمودار ۱: نمودار روند تغییرات میانگین افسردگی قبل، پایان فاز اول و پایان پیگیری در دو گروه مورد پژوهش

بحث

تحلیل یافته‌های این پژوهش گویای این مطلب است که درصد کاهش افسردگی، اضطراب و همچنین حجم ادم لنفاوی زنان مبتلا به ادم لنفاوی تحت درمان CDT گروه آزمون که آرام‌سازی عضلانی پیشرونده را طی ۹-۷ هفته به صورت روزانه انجام می‌دادند، بیش از بیماران گروه کنترل بوده. به طور کلی، روند تغییرات اضطراب، افسردگی در فاصله زمانی قبل تا پایان دوره پیگیری، نشان‌دهنده مؤثر بودن تکنیک‌های تن‌آرامی بر این متغیرها در گروه آزمون است.

پژوهش حاضر برر روی ۳۱ خانم مبتلا به لنفادم ناشی از سرطان پستان انجام شد که مشخصات جمعیتی و بالینی مشابه سایر مطالعات داشتند. میانگین و انحراف معیار سنی در گروه کنترل ۱۰/۸±۵۲ و در گروه آزمون ۱۱/۵±۵۵ سال بود. بر اساس مطالعه خوش‌نظر و همکارانش نیز میانگین سنی افراد دارای ادم لنفاوی ۹/۵±۵۳ سال بوده است و بیشترین افراد در گروه سنی ۶۰-۴۶ سال قرار داشتند (۱). میانگین شاخص توده بدنی در گروه کنترل و آزمون به ترتیب ۴/۳±۲۹ و ۴/۴±۳۰ کیلوگرم بر متر مربع می‌باشد. در این راستا طبق مطالعه موچن ۴۰٪ افراد دارای ادم لنفاوی دارای BMI بین ۳۰-۲۵ کیلوگرم بر متر مربع هستند (۲۲). بیشتر افراد شرکت‌کننده در این پژوهش در هر دو گروه متأهل بودند و طبق مطالعه حقیقت و همکارانش بیشتر افراد دارای ادم لنفاوی ۸۵/۵٪ متأهل (۲۳) و نیز طبق مطالعه شهر جردی و همکارانش نیز ۸۶/۶٪ افراد متأهل بودند (۲۴).

در این مطالعه مشاهده شد که درصد کاهش نمره افسردگی در گروه آزمون در حد معنی‌داری بیشتر از گروه کنترل بوده است. به‌عبارتی انجام آرام‌سازی عضلانی پیشرونده در گروه آزمون موجب شده است میانگین نمره افسردگی در این گروه نسبت به گروه کنترل کاهش بیشتری داشته باشد. یکی از اثرات شناخته شده آرام‌سازی عضلانی، تاثیر بر مشکلات روانی بیمار است. مایکل آنتونی و همکارانش در مطالعه‌ای دریافتند، ۱۰ هفته مداخله مدیریت استرس شناختی-رفتاری موجب کاهش افسردگی (نوع متوسط) و بهبودی در خوش بینی پس از ۳ ماه پیگیری می‌شود (۲۵) سجاد خدایی و همکاران در ایران نیز با انجام مداخله شناختی-رفتاری، کاهش میزان افسردگی را نشان دادند (۱۹). در مطالعات ذکر شده تن‌آرامی یکی از اجزای اصلی درمان بوده است. لذا با توجه به شیوع نسبتا بالای اختلالات اضطرابی و افسردگی در مبتلایان به سرطان پستان و اهمیت کنترل آن در پذیرش و تداوم درمان لنف ادم به نظر می‌رسد لازم است جهت تعیین میزان اثربخشی انجام مداخلات آرام‌سازی در این افراد مطالعات وسیع‌تری انجام شود. البته در مطالعه حاضر کاهش حجم ادم نیز در گروه آزمون که کاهش قابل توجه نمره افسردگی داشتند، بیش از گروه کنترل بود اما این تفاوت در سطح خطای ۵٪ معنی‌دار نبود. افزایش حجم

نمونه از راه‌کارهای موثر در بررسی دقیق‌تر نجوه تغییرات توام این دو پیامد در مطالعات آتی است.

نتایج نشان داد میانگین درصد کاهش نمره اضطراب در هر دو گروه مورد پژوهش کاهش یافته است که این کاهش در گروه آزمون بیش از گروه کنترل بوده است. این تفاوت در هر سه مرحله زمانی تحت مطالعه مشاهده شد، اما تنها در کل دوره در سطح خطای ۰/۰۵ این کاهش از لحاظ آماری معنی‌دار بوده است. در مطالعات محمد پدرام و همکاران (۲۶) گروه درمانی شناختی- رفتاری از طریق تن‌آرامی و تجسم موجب کاهش افسردگی و اضطراب در زنان مبتلا به سرطان پستان شد. طبق تحقیقات تاترو (۲۷) و بریج (۱۸) انجام تن‌آرامی موجب کاهش آشفتگی‌های روانی و بهبود خلق در بیماران سرطان پستان می‌شود. نتایج مطالعه استروس و همکارانش در سال ۲۰۰۵ نشان داد، انجام برنامه توانبخشی شامل؛ MLD، تمرین درمانی، آموزش تن‌آرامی و ماساژ در بیماران سرطان پستان موجب بهبود نمرات افراد در تست HADS در ۶ ماه پیگیری می شود (۲۸). در نتیجه تن‌آرامی استرس، اضطراب و افسردگی را در زنان مبتلا به سرطان پستان را کاهش می‌دهد که بخش قابل توجهی از برنامه مدیریت استرس به شیوه شناختی-رفتاری به تمرین‌های آرام‌سازی (آرام-سازی عضلانی پیشرونده) اختصاص دارد (۲۹) لذا اضافه کردن این مداخله در پروتکل درمان مبتلایان به لنف ادم با استرس بالا قابل توصیه است.

گرچه در کاهش حجم ادم لنفاوی بین دو گروه تفاوت معنی‌دار نبود، ولی همان‌طورکه نتایج نشان می‌دهد کاهش حجم در گروه آزمون بیشتر بوده است اما یافته‌های حاصل از مطالعه لودان (۱۶) و قربانی (۱۷) در سال ۲۰۱۱ نشان داد، انجام یوگا موجب کاهش حجم ادم لنفاوی و افزایش دامنه حرکتی در بیماران دارای ادم لنفاوی می‌شود. همچنین پژوهش ملکور و همکارانش در مورد تأثیر تن‌آرامی و تمرین‌درمانی بر روی زنان دارای ادم لنفاوی در سال ۲۰۱۰ نشان داد، انعطاف‌پذیری بازو، کیفیت زندگی و خلق در طی ۳ ماه بهبود می‌یابد (۱۵) از آنجا که در مطالعات مذکور درمان لنف ادم با روش CDT و نیز اندازه‌گیری حجم‌سنجی ادم انجام نشده است، نمی‌توان نتیجه‌گیری دقیق و کاملی داشت. در مطالعه حاضر کاهش حجم ادم هم‌سو با کاهش نمره اضطراب و افسردگی بود اما از نظر آماری ارتباط معنی‌داری مشاهده نمی‌شد. این فرضیه قویاً مطرح است که شاید در افراد با شدت بالاتری از اضطراب و افسردگی نسبت به شدت پایین‌تر بهبود حجم ادم قابل‌توجه باشد که به‌علت طراحی متفاوت مطالعه حاضر و محدودیت حجم نمونه نمی‌توان آنالیز را به تفکیک شدت‌های مختلف اضطراب و افسردگی انجام داد. طراحی و اجرای مطالعات آتی در این زمینه می‌تواند نتایج کاربردی در کنترل این عارضه فراهم آورد.

از نکات قوت این مطالعه طراحی خاص مطالعه در دو مقطع زمانی متفاوت جهت ورود گروه‌های آزمون و کنترل به مطالعه می‌باشد. علی‌رغم عدم امکان تصادفی‌سازی، با این طراحی امکان تماس دو گروه با هم و انتقال تجربیات را به حداقل رساندیم تا ضمن کاهش مخدوش‌گری در اجرای پژوهش، مسایل اخلاقی خاصی ناشی از عدم ارایه آرام‌سازی در گروه کنترل بر این طرح مترتب نباشد. خوشبختانه مقایسه توزیع فراوانی مشخصات جمعیتی و بالینی دو گروه تفاوت معنی‌داری نداشت، لذا می‌توان تقریباً تخصیص تصادفی افراد به گروه‌های تحت مطالعه را قایل بود. نظارت مداوم محقق مسئول در انجام مداخلات آرام‌سازی، انجام صحیح و موثر این مداخله را امکان‌پذیر نمود. ضمن اینکه اندازه‌گیری پیامدها توسط فرد مستقلی که در درمان دو گروه دخیل نبود، تورش مشاهده‌گر را به حداقل می‌رساند. مسلماً اختصاص زمانی برای تکمیل پرسشنامه‌های لازم، افزایش طول مدت درمان در صورت آرام‌سازی و عدم تکمیل دوره درمان توسط برخی بیماران از محدودیت‌هایی بود که پژوهشگران تلاش نمودند ضمن جلوگیری از مداخلات سوگرایانه و در صورت لزوم با جایگزینی نمونه‌ها به کنترل درآورند.

نتیجه گیری

نتایج این پژوهش مؤید تأثیر تن‌آرامی به صورت آرام‌سازی عضلانی پیشرونده روی بیماران مبتلا به ادم لنفاوی تحت درمان CDT در کاهش اضطراب و افسردگی وتاحد کمی در حجم ادم لنفاوی آنان می باشد.با توجه به نتایج این مطالعه ، به نظر می رسد که برنامه‌ریزی لازم جهت توسعه تکنیک‌های درمان لنف‌ادم و آرام‌سازی در سیستم بهداشتی و چارچوب برنامه‌ریزی آموزشی دانشجویان و درمانگران شاغل در مراکز درمانی می‌تواند در بهبود کیفیت زندگی این بیماران مؤثر باشد. بررسی میزان تاثیر و نیز هزینه اثربخشی ادغام روش‌های مختلف درمانی

مشکلات روان‌شناختی با درمان لنف‌ادم، علی‌الخصوص در افراد با شدت بالای این اختلالات، از موضوعات ارزشمندی است که لازم است در تحقیقات گسترده‌تری مورد ارزیابی قرار گیرند.

تشکر و قدردانی

در پایان لازم می‌دانیم از بیماران و کارکنان محترم کلینیک توانبخشی سیدخندان که همکاری لازم با پژوهشگران را دریغ ننمودند، سپاسگزاری کنیم.

References

۱. خوش‌نظر طاهره‌السادات، محمدی فرحناز، خانکه حمیدرضا، حقیقت شهپر. تأثیر توانبخشی مبتنی بر منزل بر میزان ادم لنفاوی و فعالیت روزانه دست در زنان بعد از ماستکتومی مراجعه‌کننده به مرکز بیماری‌های پستان جهاددانشگاهی واحد علوم پزشکی تهران در سال ۱۳۸۸. فصلنامه بیماری‌های پستان ایران ۱۳۸۹؛ ۳(۳ و ۴): ۴۲-۳۴.

۲. حقیقت شهپر، اکبری محمد اسماعیل، غفاری شهرام، یاوری پروین. محاسبه بار مالی اقدامات تشخیصی و درمانی اولیه سرطان‌های غیرمتاستاتیک پستان در ایران ۱۳۹۱. فصلنامه بیماری‌های پستان ایران ۱۳۹۲؛ ۴(۱و۲): ۲۵-۱۴.

۳. فلاح راحله، گلزاری محمود، داستانی محبوبه، ظهیرالدین علیرضا، موسوی سیدمحمد مهدی، اکبری محمد اسماعیل، اثر بخشی مداخله معنوی به شیوه گروهی بر ارتقا امید و سلامت روان در زنان مبتلا به سرطان پستان. مجله اندیشه و رفتار ۱۳۹۰؛۵ (۱۹): ۷۶-۶۵.

۴. بختیاری مریم، اسلامی محمد، فشارکی محمد، کوشا سهیلا، بررسی تأثیر آموزش مراقبت از خود بر سلامت روان زنان مبتلا به سرطان پستان کاندید شیمی‌درمانی مراجعه‌کننده به بیمارستان امام خمینی(ره) تهران ۱۳۹۰. فصلنامه بیماری‌های پستان ۱۳۹۰؛ ۴ (۱و۲)، ۷-۴۲.

۵. سجادیان اکرم‌السادات، حقیقت شهپر، منتظری علی، کاظم نژاد انوشیروان، علوی افسانه، سازگاری در بیماران مبتلا به سرطان پستان قبل و بعد از درمان. فصلنامه بیماری‌های پستان ایران ۱۳۹۰؛ ۴ (۳)، ۸-۵۲ .

6. Tam E, SheL, Munnek J. Clinician awareness and knowledge of breast cancer related lymphedema inlarge, integrated health care dliverysetting. Breast cancer retreat 2011;1029-38.

7. Martin M, Hernandz M, Avendan C, Rodriquez F, Martinez H. Manual lymphatic drainage in paitient with breast cancer related lymphedema. Biomed central 2011.

8. Disipio T, Rye S, Newman B, Hayes S. Incidence of unilateral arm lymphedema after breast cancer: a systematic review and meta-analysis. Lacentoncol 2013;14.

9. Shier B. The occupational therapist’s role in lymphedema self-management. Occupational Therapy Now 2012; 14(3): 19-21

10. Ridner SH. The psychosocial effect of lymphedema. lymphatic research & biology 2009; 2(7).

۱۱. لطفی معصومه. ادم لنفاوی. فصلنامه بیماری‌های پستان ایران ۱۳۸۷؛ ۱(۱): ۳-۵۰.

12. Erikson V, Pearson M, Ganz P, Adams J, Kahn K. Arm edema in breast cancer patients. Journal of national cancer institute 2001; 93(2): 96-111.

13. Vignes S, Porcher R, Arrault M, Dupuy A. Longterm management of breast cancer–related lymphedema after intensive decongestive physiotherapy. Breast cancer research and treatment 2007; 101(3): 285-90.

14. Payne R. Relaxation techniques a practical handbook for the health care professional, second edition. Churchill livingstone 2000: 3-36.

15. Mcclure M, Mcclure R, Day R, Brufsky A. Randomize control trail of the breast cancer recovery program for women with breast cancer related lymphedema. American journal of occupational therapy 2010; 64(1): 59-72

16. Loudon A, Barnett T, Piller N, Immink M, Visentin D, Williams A.The effect of yoga on women with secondary arm lymphedema from breast cancer treatment .

BMC Complementary and Alternative Medicine 2012; 12: 66

۱۷. قربانی مریم، سخنگویی یحیی. تأثیر دو روش تمرینی پیلاتس و یوگا بر دامنه حرکتی و درد اندام فوقانی در زنان مبتلا به سرطان پستان پس از جراحی سرطان پستان. فصلنامه بیماری‌های پستان ایران ۱۳۹۱؛ ۵ (۲و۳): ۹۲-۸۴.

18. Bridge L, Benson P, Pietroni P, Priest R. Relaxation and imagery in the treatment of breast cancer. BMJ 1988; 297:1169-72.
19. Khodaei S, Dastjerdi R, Haghighat F, Saadatjo A, KeramatiA. The impact of cognitive behavioral group therapy for depression in patients with cancer (in Persian). Birjand University of Medical Sciences 2011; 17(3): 183-90.
20. Lawenda BD, Mondry TE, Johnstne PAS. lymphedema. A primer on the identification and management of a chronic condition in oncologic treatment. Cancer journal for clinicians 2009; 59(1).
21. Montazeri A, Vahdaninia M, Ebrahimi M, Jarvandi S. The Hospital Anxiety and Depression Scale (HADS): translation and validation study of the Iranian version. Health and Quality of Life Outcomes 2003; 1(14).
22. Moschen R, Kemmler G, Schweigkofler H, et al. Use of alternative/complementary therapyin breast cancer patients–psychological prespective.Support care cancer 2001; 9:267-74.
23. Haghighat SH, Lotfi M, Yunesian M, Akbari M, Nazemi F, Weiss J. Comparing two treatment methods for post mastectomy lymphedema: complex decongestive therapy alone in combination with intermittent pneumatic compression. Lymphology 2010; 43:25-33.

۲۴. شهرجردی شهناز، درویش شفیقی صدیقه، تأثیر ۵ هفته ماساژ بر میزان استرس و افسردگی زنان بعد از عمل جراحی سرطان پستان. فصل‌نامه بیماری‌های پستان ایران ۱۳۹۲؛ ۶(۳):۴۶-۳۷.

25. Antoni M, Lehman J, Klibourn K, Boyers A, Culver J, Alferi S, et al. Cognitive behavioral stress management intervention on decress the prevalence of depression and enhances benefit finding among women under treatment for early stage breast cancer. Journal health psychological 2001; 20(1).

۲۶. پدرام محمد، محمدی مسعود، نظیری قاسم، آیین-پرست ندا. اثر بخشی گروه درمانی شناختی- رفتاری بر درمان اختلال اضطراب، افسردگی و ایجاد امیدواری در زنان مبتلا به سرطان سینه. فصلنامه علمی پژوهشی زن، جامعه ۱۳۸۹؛ ۱ (۴)، ۷۶-۶۱.

27. Tatrow K, Montgomery K. Cognitive behavioral therapy techniques for distress and pain in breast cancer patient :a meta analysis. Journal of behavioral medicine 2006; 29(1).
28. Strauss- blasche G, Griad E, Ekmekcioglu C , Hlandschik B, Marktl W. Combined inpatient rehabilitation and spa therapy for breast cancer patients. Cancer nursing 2005; 70128(5).

۲۹. صفرزاده عطیه، روشن رسول، شمس جلال‌الدین. اثر بخشی آموزش مدیریت استرس به شیوه شناختی-رفتاری در کاهش اضطراب، استرس و افسردگی زنان مبتلا به سرطان پستان. فصلنامه مطالعات روان‌شناسی بالینی ۱۳۹۱؛ ۲: ۶.
